# Modifying lignin to improve bioenergy feedstocks: strengthening the barrier against pathogens?^†^

**DOI:** 10.3389/fpls.2013.00070

**Published:** 2013-04-05

**Authors:** Scott E. Sattler, Deanna L. Funnell-Harris

**Affiliations:** ^1^Grain Forage and Bioenergy Research Unit, Agricultural Research Service - United States Department of AgricultureLincoln, NE, USA; ^2^Department of Agronomy and Horticulture, University of Nebraska at LincolnLincoln, NE, USA; ^3^Department of Plant Pathology, University of Nebraska at LincolnLincoln, NE, USA

**Keywords:** plant pathogens, lignin, *brown midrib*, monolignol pathway, CAD, COMT

## Abstract

Lignin is a ubiquitous polymer present in cell walls of all vascular plants, where it rigidifies and strengthens the cell wall structure through covalent cross-linkages to cell wall polysaccharides. The presence of lignin makes the cell wall recalcitrant to conversion into fermentable sugars for bioenergy uses. Therefore, reducing lignin content and modifying its linkages have become major targets for bioenergy feedstock development through either biotechnology or traditional plant breeding. In addition, lignin synthesis has long been implicated as an important plant defense mechanism against pathogens, because lignin synthesis is often induced at the site of pathogen attack. This article explores the impact of lignin modifications on the susceptibility of a range of plant species to their associated pathogens, and the implications for development of feedstocks for the second-generation biofuels industry. Surprisingly, there are some instances where plants modified in lignin synthesis may display increased resistance to associated pathogens, which is explored in this article.

## INTRODUCTION

**FIGURE 1:**
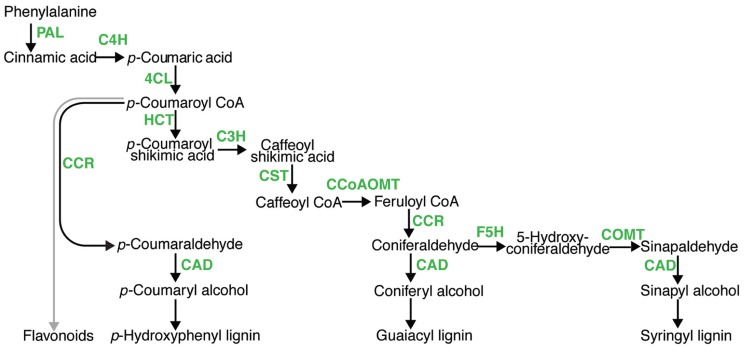
**A model for monolignol pathway.** In phenylpropanoid metabolism, there are 10 enzymatic steps (green) leading to hydroxycinnamyl alcohols which are polymerized into lignin, namely; phenylalanine ammonia lyase (PAL), cinnamate 4-hydroxylase (C4H), 4-coumarate-CoA ligase (4CL), hydroxycinnamoyl CoA:shikimate transferase (HCT), *p*-coumarate 3-hydroxylase (C’3H), caffeoyl CoA *O*-methyltransferase (CCoAOMT), cinnamyl CoA reductase (CRR), ferulate 5-hydroxylase (F5H), caffeic acid *O*-methyltransferase (COMT), and cinnamyl alcohol dehydrogenase (CAD).

In the U.S. and around the world, there are increasing efforts to develop and utilize alternatives to fossil fuels to meet our energy needs, thereby reducing carbon dioxide emissions that potentially impact global warming. Currently, corn grain and sugarcane juice are being converted into ethanol for blending in gasoline. Research efforts have been directed toward developing means to convert plant biomass from a range of sources into liquid fuels for the transportation sector. Cellulosic biofuels rely on chemically and biochemically breaking down cell wall polysaccharides (cellulose and hemicellulose) into their sugar monomers, and converting the sugar into fuels. A third component of cell walls is the phenolic polymer lignin, which structurally fortifies the cell walls making them rigid and resistant to microbial degradation. Lignin content has been shown to negatively impact cellulosic bioenergy conversion via saccharification and fermentation to ethanol (Chen and Dixon, 2007; Dien et al., 2009), which has made reducing lignin and altering lignin composition a major target to improve plants for cellulosic bioenergy. Conversely, increasing the lignin content of herbaceous feedstocks may benefit conversion of biomass to syngas and bio-oil biofuel via pyrolysis. In either case, efforts to manipulate lignin content and composition have primarily focused on the 10 steps of the monolignol pathway (**Figure 1**), in which lignin monomers are synthesized from the amino acid phenylalanine, then oxidatively polymerized into hydroxyphenol- (H-), guiacyl- (G-), or sinapyl- (S-) lignin. Lignin serves the critical function of reinforcing vascular elements for water transport under negative pressure; in severely lignin deficient plants, vascular collapse has been observed (Piquemal et al., 1998; Jones et al., 2001; Ruel et al., 2009). Thus, there is a lower limit for lignin manipulation. In addition to its roles in fortifying cell walls, lignin deposition has long been implicated as an important defense mechanism against pests and pathogens (Vance et al., 1980). A critical question for bioenergy feedstock development is whether manipulating lignin content and composition will be detrimental to plant defenses against pathogens. Herein, we examine this question and the cause for concern in manipulating lignin, based on current published literature.

## ROLE OF LIGNIN IN PLANT DEFENSE

There is a strong case for the involvement of lignin in plant defense. Lignin provides a physical barrier against initial ingress (Buendgen et al., 1990; Bonello et al., 2003), and in a wide range of plant species lignin or lignin-like phenolic polymers are induced and rapidly deposited in cell walls in response to both biotic and abiotic stresses, which may limit further growth and/or confine invading pathogens (Siegrist et al., 1994; Lange et al., 1995; Baayen et al., 1996; Smit and Dubery, 1997; Bonello et al., 2003; Hudgins et al., 2004; Wuyts et al., 2006; Menden et al., 2007). “Defense” lignin may prevent further ingress or diffusion of pathogen-produced toxins (Carver et al., 1994; Duschnicky et al., 1998). However, “defense” lignin deposition is often only monitored microscopically as cell wall autofluorescence or via histochemical staining techniques (Haegi et al., 2008; Eynck et al., 2009). “Defense” lignin was often shown to have elevated levels of H-subunits as compared to structural lignin in the cases analyzed (Ride, 1975; Hammerschmidt et al., 1985; Doster and Bostock, 1988; Robertsen and Svalheim, 1990; Lange et al., 1995). The phenylpropanoid pathway leads to the synthesis of numerous other phenolic compounds besides monolignols, including phenolic phytoalexins, stilbenes, coumarins, and flavonoids (Lo and Nicholson, 1998; Yu et al., 2000, 2005; Dixon et al., 2002). A number of these compounds have also been implicated in plant defense (Weiergang et al., 1996; Dicko et al., 2005; Lozovaya et al., 2007). For example, the defense signaling hormone salicylic acid (SA) might also be derived from the phenylpropanoid pathway in some plants (Ruuhola and Julkunen-Tiitto, 2003; Pan et al., 2006). Moreover, abiotic or biotic stresses including pathogens have been shown to induce the expression of genes encoding monolignol biosynthetic enzymes in many plant species (Kliebenstein et al., 2002; Truman et al., 2006; Olsen et al., 2008; Zhao et al., 2009). Likewise, the protein levels and enzymatic activities corresponding to these genes were also shown to be elevated under these stresses in a number of plant species (Mitchell et al., 1999). Together these observations indicate that lignin deposition is part of a generalized resistance response to biotic stresses (Nicholson and Hammerschimdt, 1992). Thus, it remains to be determined whether bioenergy crops that are impaired or altered in their ability to synthesize lignin will also be impaired in their ability to induce these defense responses upon pathogen attack. Recent research has suggested that impairing lignin biosynthesis does not lessen resistance to some pathogens (Delgado et al., 2002; Funnell and Pedersen, 2006; Peltier et al., 2009; Quentin et al., 2009; Funnell-Harris et al., 2010). Because very little has been published on the effects of lignin modification on plant–pathogen interactions in bioenergy feedstocks with the exception of maize (*Zea mays*) and sorghum (*Sorghum bicolor*), we have also included a review of the literature on effects of impairing steps in the monolignol pathway to pathogen responses in other plant species.

However, several pathogens have been isolated and identified that pose potential threats to some of the perennial grass species being considered as herbaceous bioenergy feedstocks including switchgrass (*Panicum virgatum*), napiergrass (*Pennisetum purpureum*), sugarcane/energycane (complex hybrid *Saccharum* spp.), and miscanthus (*Miscanthus *× *giganteus*). Pathogens may pose a greater threat to perennial grasses as compared to annual row crops such as maize and sorghum, because production relies on establishment and harvest across multiple years before replanting, and the continual presence of the plants in the field provides refuge for the pathogens. Another factor that could impact plant–pathogen interactions is the level of genetic diversity within the field setting. Switchgrass varieties are maintained as an outcrossing population (Martïnez-Reyna and Vogel, 2002; Nageswara-Rao et al., 2012), hence maintain level genetic diversity. In contrast, the clonally propagated miscanthus is genetically identical (Lewandowski et al., 2000). Fungal leaf rusts caused by *Puccinia* spp. have been identified in sugarcane (Dixon et al., 2010) and switchgrass (Gustafson et al., 2003; Wadl et al., 2011). Fungal leaf blights caused by *Leptosphaerulina chartarum* (Ahonsi et al., 2013) and *Leptosphaeria *sp*. *(O’Neill and Farr, 1996) have been identified in *Miscanthus* × *giganteus *or related *Miscanthus *species. Rhizome rot is a significant threat to miscanthus establishment, which is caused by three pathogenic fungal species (*Fusarium avenaceum*, *F. oxysporum*, and *Mucor hiemalis*; Beccari et al., 2010; Covarelli et al., 2012). A fungal smut has been identified on napiergrass caused by *Ustilago kamerunensis *(Farrell et al., 2001). Anthracnose, a foliar blight caused by *Colletotrichum navitas* has also been identified in switchgrass (Crouch et al., 2009). Overall, these studies indicated that similar to row crops, fungal pathogens pose a serious threat to these bioenergy crops. In addition, viruses also pose a threat to bioenergy feedstocks, which have been documented in switchgrass and miscanthus (Chatani et al., 1991; Turina et al., 1998; Lamptey et al., 2003).

## PHENYLALANINE AMMONIA LYASE

Phenylalanine ammonia lyase (PAL) is the first committed step in monolignol biosynthesis and the phenylpropanoid pathway. Altering the expression of this central gene has been shown to impact plant–pathogen interactions in model systems. In tobacco (*Nicotiana tabacum*), antisense suppression of this gene led to increased susceptibility to the fungal pathogen *Cercospora nicotianae*, the causal agent of frogeye disease (Maher et al., 1994; Shadle et al., 2003). Tobacco plants over-expressing this gene had reduced susceptibility to *Cercospora nicotianae*, but resistance to tobacco mosaic virus (TMV) was unchanged (Shadle et al., 2003). Over-expression of PAL in tobacco resulted in significantly increased levels of the defense signaling compound SA and the defense related compound chlorogenic acid upon induction (Howles et al., 1996; Felton et al., 1999). Furthermore, over-expressing the bacterial salicylate hydroxylase gene (NahG), which degrades SA, in concert with PAL over-expression, increased susceptibility to TMV although resistance to *Cercospora nicotianae *was unaffected (Shadle et al., 2003). These results indicated that TMV resistance required SA but not chlorogenic acid, while increased resistance to *Cercospora nicotianae *only required elevated levels of chlorogenic acid and not SA (Shadle et al., 2003). Conversely, PAL over-expression in tobacco resulted in increased susceptibility to the insect *Heliothis virescens*, and a PAL-suppressed line had increased resistance, which was attributed to the antagonistic relationship between SA signaling and jasmonic acid (JA) signaling (Felton et al., 1999). The *Arabidopsis* genome contains four PAL genes. T-DNA insertion mutants were isolated for all four genes, and these mutants were crossed to create double, triple, and quadruple *pal* mutants (Huang et al., 2010). The *pal1/2/3/4* quadruple mutant showed increased susceptibility to the bacterial pathogen *Pseudomonas syringae *relative to WT, and *pal1/2* also had increased susceptibility to this pathogen relative to WT and intermediate to *pal1/2/3/4 *(Huang et al., 2010). SA, lignin and anthocyanin related pigment levels were significantly reduced in *pal1/2/3/4* plants (Huang et al., 2010). However, these changes in susceptibility to pathogens cannot be directly attributed to changes in lignin content or composition, because PAL is involved in the synthesis of the full range of phenolic compounds, some of which have been implicated in defense, like chlorogenic acid and flavonoid compounds.

## HYDROXYCINNAMOYL CoA: SHIKIMATE TRANSFERASE

In *Arabidopsis* (*Arabidopsis thaliana*) and alfalfa (*Medicago sativa*), antisense/RNAi suppression of hydroxycinnamoyl CoA: shikimate transferase (HCT) one of the initial steps in monolignol biosynthesis, illustrates the potential of genetic/transgenic alterations to this pathway to constitutively activate defenses (Gallego-Giraldo et al., 2011a, b). In both plant species, antisense/RNAi suppression of HCT resulted in significant reductions in lignin content and stunted plants relative to WT (Shadle et al., 2007; Li et al., 2010). In alfalfa, these plants showed increased resistance to the fungal pathogen *Colletotrichum trifolli *(Gallego-Giraldo et al., 2011b). In the absence of a pathogen, SA levels were highly elevated relative to WT in both species and several defense related genes were also highly induced relative to WT in alfalfa (Gallego-Giraldo et al., 2011a, b). In *Arabidopsis*, growth was partially restored in NahG HCT-RNAi and *SA induction deficient2-2*(*sid2-2*) HCT-RNAi; *SID2* encodes an isochorismate synthase required for isochorismate-dependent SA synthesis. These results indicate that the stunted growth phenotype is due to elevated SA, occurring through an isochorismate-dependent pathway, rather than resulting from excess phenylalanine intermediates leading to the synthesis of SA in HCT-RNAi plants. Elevated levels of cold-water extractable pectin were correlated to elevated SA levels in transgenic alfalfa plants, which were RNAi-suppressed for six different genes (steps) in the monolignol pathway (Gallego-Giraldo et al., 2011a). Highest levels of SA and cold-water extractable pectin were observed in HCT-suppressed lines relative to WT or the other five monolignol biosysthetic gene-suppressed lines (Gallego-Giraldo et al., 2011a). Pectic oligosaccharides have been implicated as defense signals in other systems (Darvill and Albersheim, 1984; Roco et al., 1993), and are the potential trigger for the defense responses observed in HCT lines. Thus, the effects observed in the HCT-suppressed lines could potentially result from changes in cell wall structure, the first line of defense for the plant, rather than directly resulting from an alteration in phenylpropanoid metabolism.

## CAFFEIC *O*-METHYLTRANSFERASE

In *Arabidopsis* and tobacco, antisense/RNAi suppression of caffeic *O*-methyltransferase (COMT), the penultimate step in monolignol biosynthesis, was reported to increase resistance to pathogens or to have no effect on interaction with pathogens. In *Arabidopsis*, *comt1* mutants show enhanced resistance to the oomycete pathogen *Hyaloperonospora arabidopsidis*, which is the causal agent of downy mildew (Quentin et al., 2009). There were significantly fewer asexual spores on *comt1* plants relative to WT, because sexual sporulation was increased in *comt1* plants, resulting in attenuated mycelium growth (Quentin et al., 2009). Exposing the pathogen to the phenolic compound 2-*O*-5-hydroxyferuloyl-L-malate, which is present in *comt1* and absent in WT plants, promoted sexual reproduction (Quentin et al., 2009). However, *comt1* plants showed increased susceptibility relative to WT to the bacterial pathogens *Xanthomonas campestris pv. campestris *and *Pseudomonas syringae *and a less virulent strain (T4) of the fungal pathogen *Botrytis cinerea *(Quentin et al., 2009). In tobacco, COMT antisense lines were resistant to *Agrobacterium tumefaciens *infection, and had reduced tumor area and mass relative to WT (Maury et al., 2010). Bacterial virulence (vir) gene induction was reduced in the COMT-suppressed line likely due to the highly reduced level of the phenolic elicitor of *Agrobacterium *acetosyringone (Maury et al., 2010). Acetosyringone is probably derived from Coenzyme A dependent β-oxidation of hydroxycinnamoyl-CoA intermediates of monolignol biosynthesis (Blount et al., 2002; Negrel and Javelle, 2010). In *Arabidopsis* and tobacco, the alteration to phenylpropanoid metabolism by reducing COMT activity appears to directly result in increased resistance to two of the pathogens tested, downy mildew and *Agrobacterium*, respectively. However, the same *Arabidopsis* plants showed increased susceptibility to two bacterial pathogens and a less virulent strain of *Botrytis cinerea*.

## CINNAMYL ALCOHOL DEHYDROGENASE

In flax (*Linum usitatissimum* L.), RNAi suppression of the cinnamyl alcohol dehydrogenase (CAD) gene, the last step in monolignol biosynthesis, increased susceptibility to the pathogenic fungus *F. oxysporum*. A seedling assay showed the percent of infected seedlings was twofold higher in two CAD RNAi lines relative to WT (Wróbel-Kwiatkowska et al., 2007). In *Arabidopsis*, the *cad-c* and *cad-d* double mutants, which were shown to be required for monolignol biosynthesis (Kim et al., 2004; Sibout et al., 2005), showed increased susceptibility to both a virulent and an avirulent strain of the bacterial pathogen *Pseudomonas syringae* pv. *tomato* (Pst;(DC3000, virulent; DC3000/avrPphB, avirulent)) relative to WT based on bacterial growth following inoculation (Tronchet et al., 2010). Together, these results suggest that CAD-deficiency may increase the susceptibility of plants to a range of pathogens. This result might have implications for bioenergy feedstocks, because CAD suppression is often targeted to reduce lignin content.

## OTHER STEPS IN MONOLIGNOL SYNTHESIS

In *Arabidopsis*, the *ferulic acid 5-hydroxylase 1* (*fah1*) mutant, which encodes the ferulic acid 5-hydroxylase (F5H), last hydroxylase in monolignol synthesis, showed increased susceptibility to the fungal pathogen *Sclerotinia sclerotiorum* relative to WT in leaf assays (Huang et al., 2009). In diploid wheat (*Triticum monococcum* L.), five genes in monolignol biosynthesis were transiently silenced using particle bombardment of an RNAi vector containing PAL, caffeoyl-CoA *O*-methyltransferase (CCoAMT), F5H, COMT, or CAD genes (Bhuiyan et al., 2009). The bombarded leaves were inoculated with the powdery mildew fungal pathogens *Blumeria graminis* f. sp. *tritici* (host-specific) and *Blumeria graminis* f. sp. *hordei* (non-host). The silencing of all five genes individually and in pairs increased the susceptibility to both pathogens relative to the control bombarded with the empty RNAi vector, as determined by penetration efficiency of the fungus (Bhuiyan et al., 2009). However, it is unclear whether this transient approach to gene silencing is relevant to the stable approaches used to impair genes within this pathway for bioenergy feedstock improvement.

## BIOENERGY FEEDSTOCKS

There has been very little published on plant pathogen interactions in bioenergy feedstocks with modified lignin content and composition. In hybrid poplar (*Populus tremula *× *Populus alba*), it has been reported that no increased disease incidence were observed in antisense COMT or CAD lines relative to WT (Halpin et al., 2007). The one exception where the effects of lignin modification on plant pathogen interactions has been examined are the *brown midrib *(*bmr/bm*) mutants of sorghum and maize (*Zea mays*), which have long been known to have reduced lignin content (Jorgenson, 1931; Porter et al., 1978). There are at least five *Bm* loci identified in maize (Chen et al., 2012) and at least seven *Bmr* loci in sorghum (Pedersen et al. unpublished). Three *Bmr* loci have been cloned and characterized in sorghum. *Bmr2*, *Bmr6*, and *Bmr12* all encode enzymes in monolignol biosynthesis: a 4-coumarate coenzyme A ligase (4CL), a CAD, and a COMT, respectively (Bout and Vermerris, 2003; Saballos et al., 2009, 2012; Sattler et al., 2009). In maize, the *Bm3* locus encodes a COMT protein (Vignols et al., 1995) that is orthologous to *Bmr12*, and the *Bm1 *locus encodes a CAD protein that is orthologous to *Bmr6 *(Halpin et al., 1998; Chen et al., 2012). Lignin deposition and the induction of phenylpropanoid-related genes during pathogen attack (described above) led to the assumption that *brown midrib* plants are inherently more disease susceptible when challenged. However, studies examining both grain and stalk fungal pathogens, which are the most prevalent and economically significant sorghum pathogens (Chandrashekar and Satyanarayana, 2006), have in general indicated the contrary.

Fungal infection of *bm*/*bmr* grain may not appear to be relevant to bioenergy, however, fungal infection of grain can impair seed germination (Raju et al., 1999; Prom et al., 2003), which is critical for all cropping systems. Under field conditions without inoculation, maize *bm3* grain showed significantly increased colonization by members of the *Gibberella fujikuroi *fungal species complex as compared to WT grain (Nicholson et al., 1976). In contrast, studies using uninoculated field-grown sorghum showed that *bmr6 *and *bmr12 *grain had the same level of colonization or significantly reduced fungal colonization relative to WT, which included the sorghum pathogen *F. hapsinum*, a *G. fujikuroi *species complex member (Funnell and Pedersen, 2006; Funnell-Harris et al., 2010). Other *Fusarium *spp. colonized both *bmr6* and *bmr12* grain at similar levels or significantly reduced colonization relative to WT (Funnell and Pedersen, 2006; Funnell-Harris et al., 2010). **In particular, two species that commonly infected WT grain were significantly reduced or absent in *bmr12 *grain, *F. proliferatum *and a member of the *F. incarnatum-F. equiseti *species complex (O’Donnell et al., 2007), respectively (Funnell-Harris et al., 2010). Taken together, these results indicated that impairing CAD or COMT activity in sorghum did not increase susceptibility to these *Fusarium *spp., and *bmr12 *grain restricted or excluded colonization of two species. These results contradict the single early report from maize where *bm3* grain, which is also COMT-deficient, showed increased colonization by the *G. fujikuroi *species complex (Nicholson et al., 1976).

Studies examining the susceptibility of maize and sorghum *bm/bmr* mutants to stalk rot pathogens, which impact biomass quality and can contribute to lodging, also showed no change in resistance or increased resistance relative to WT, similar to the grain studies. *F. thapsinum* was inoculated in the peduncles of *bmr6*, *bmr12* and WT from six near-isogenic backgrounds and disease severity was determined by the length of the purple disease lesion resulting from the fungal infection. Lesion lengths were significantly shorter than corresponding WT background for many *bmr6* and *bmr12* lines, and lesion lengths were significantly shorter than WT for one or both *bmr* lines across four different genetic backgrounds (Funnell and Pedersen, 2006; Funnell-Harris et al., 2010). There were no cases where the lesion length was significantly greater in a *bmr* line relative to the corresponding WT line (Funnell and Pedersen, 2006; Funnell-Harris et al., 2010). Peduncle inoculations of *bmr6*, *bmr12*, **and wild-type lines with four *Fusarium *species and *Alternaria alternata* consistently resulted in decreased lesion lengths on one or both *bmr *mutants relative to WT for the following pathogens; *F. thapsinum*, *F. verticillioides* and *Alternaria* *alternata *(Funnell-Harris et al., 2010). Overall, these results consistently indicated that *bmr6* and *bmr12* were not more susceptible to these pathogens than WT, and in some cases the two *bmr *mutants appeared to be more resistant to specific pathogens relative to WT. However, fungal viability was assessed within the lesions and outside the lesions, and fungal growth was detected within and outside borders of lesions from *bmr12* inoculated peduncles (Funnell-Harris et al., 2010). This result suggests that fungal growth is greater in healthy-appearing tissues outside the necrotic, discolored tissue defined as the “lesion” in *bmr12* plants, although these lesions were similar in size or significantly shorter than WT in *bmr12* peduncles. Nevertheless, CAD or COMT deficiency in sorghum does not appear to significantly increase susceptibility of plants to these stalk rot pathogens.

A study using inoculations of another fungal stock pathogen *Macrophomina phaseolina*, which causes charcoal rot, also demonstrated *brown midrib* mutants were not more susceptible to this pathogen. *bmr* mutants from sorghum (*bmr2*, *bmr6*, *bmr7*, *bmr12*, *bmr26*, and *bmr28*; three loci, *bmr28* is allelic to *bmr6*, and *bmr7* and *bmr26* are allelic to *bmr12*; Saballos et al., 2008) and maize (*bm1*, *bm2*, *bm3*, and *bm4*; four loci) were inoculated with *Macrophomina phaseolina* and lesion lengths were compared to corresponding WT lines (Tesso and Ejeta, 2011). Lesion lengths were not significantly different between *bm/bmr* mutants and the corresponding WT backgrounds for both maize and sorghum (Tesso and Ejeta, 2011). Stalk strength as determined using rind penetration resistance was significantly reduced in maize *bm* mutants relative to WT (Tesso and Ejeta, 2011). Interestingly, reduced mechanical stalk strength did not appear to increase susceptibility (Tesso and Ejeta, 2011). However, all studies relied on artificial inoculation to ensure a consistent disease response. If decreased rind penetration resistance (stalk strength) increases the ability of the fungi to initially enter and penetrate the stalk, then results from these studies may be misleading. All the *bm/bmr* mutants examined resulted in similar susceptibility to the charcoal rot pathogen, even though at least three different steps in monolignol biosynthesis were impaired by the corresponding *bmr* mutation; 4CL (*bmr2*), COMT (*bm3/bmr12*), and CAD (*bm1/bmr6*). The general trend from these studies indicate that maize and sorghum *brown midrib *mutants are not more susceptible to stalk rot pathogens, and in some cases show increased generalized resistance to specific pathogens.

There are several explanations for the instances of increased generalized resistance observed in the *brown midrib* mutants. Although the ability of these *bmr* plants to synthesize structural lignins is decreased and/or altered, there is no evidence *bmr* plants are impaired in their ability to synthesis “defense” lignin in response to pathogen attack, and the response might even be enhanced. Another explanation is that blocking a step in the lignin biosynthetic pathway would cause accumulation of lignin precursors and other phenolic compounds, because additional substrates would be available for their synthesis. Indeed it has been shown that some of these precursors inhibit the growth of pathogenic fungi or inhibit production of virulence factors (Dowd et al., 1997; Hua et al., 1999; McKeehen et al., 1999; Beekrum et al., 2003). For example, accumulation of ferulic acid, *p*-coumaric acid, and sinapic acid has been correlated with resistance to *Fusarium *spp*.* (McKeehen et al., 1999; Siranidou et al., 2002). We have observed increased soluble phenolic compounds in *bmr6* and *bmr12* plants relative to WT (Palmer et al., 2008). Alternatively, perturbing the synthesis of lignin, a component of the cell wall which is the first line of defense against pathogens, could trigger generalized cell wall based defense responses similar to HCT-RNAi lines in *Arabidopsis* and alfalfa (Gallego-Giraldo et al., 2011a). A review focused on the broader role of the cell wall in plant defense was previously published (Underwood, 2012), which documents the significance of the plant cell wall in responses to a wide range of pathogens.

## PROSPECTIVE

These studies from a variety of plants indicate that reducing lignin content and altering its composition will not inevitably increase the susceptibility of bioenergy feedstocks to pathogens. There were not any clear trends that indicate that impairing a specific step in monolignol biosynthesis would affect plant susceptibility. In fact, studies from sorghum and maize indicate that impairing CAD or COMT activity in these lignin-modified plants showed more resistance to specific fungal pathogens, albeit these plants are not as resistant to the pathogen as resistant plant germplasm used in breeding efforts. In bioenergy feedstock species, modifications to monolignol biosynthesis will need to be evaluated on a case by case basis to determine the impact of pathogen susceptibility.

## Conflict of Interest Statement

The authors declare that the research was conducted in the absence of any commercial or financial relationships that could be construed as a potential conflict of interest.
